# On the Resuscitation of Apparently Still-Born Children

**Published:** 1827-08

**Authors:** J. Toogood

**Affiliations:** Member of the Royal College of Surgeons, London.


### MIDWIFERY.

### *On the Resuscitation of apparently Still-born Children.* By J. Toogood, Esq. Member of the Royal College of Surgeons, London.

Having recently met with a case of an apparently still-born child, which was recovered after an unusual length of time, I am induced to lay an account of it before the public, because, from my experience in many cases, I believe a very large proportion of children, apparently dead-born, may be resuscitated, if proper means be resorted to, and persevered in for a sufficient length of time. But the modes generally employed to restore life, such as immersing the infant in warm water, friction, and pouring stimulants down the throat, are not at all calculated to produce the effect intended; and, if these means do not succeed after a short trial, all further attempts are generally abandoned. The plan I always adopt, which has never failed where the child was living during the birth, is very simple, and only requires perseverance. I hope, therefore, I shall not be occupying the time of your readers unnecessarily by detailing some successful cases, under circumstances by no means favourable, which I have selected from a great many more.

Grace White, a very weakly woman, far advanced in consumption, was seized in the morning with uterine hemorrhage, which continued slightly till the evening, when I saw her; and, whilst standing by her bed-side, the flooding increased with such violence that I thought it best to deliver her instantly. The child was still-born. As soon as I had removed it from the mother, and seen her safe from any immediate danger, I placed a napkin over the child's mouth, and inflated its lungs from my mouth, pressing out the air from the chest afterwards, and thus imitating natural respiration. After having continued this process for thirty-five minutes, the child made a very slight attempt to breathe, and the face became slightly suffused; by persevering ten minutes longer, the free action of the lungs was established, and the child cried lustily.

The next case was that of a poor woman, named Sarah Holmes, of the parish of Spaxton, who had been in labour a long time with a presentation of the arm, and, as it was her first confinement, it became very difficult to turn the child, particularly as she was advanced in age, and the parts were very rigid. The child was still-born; but, by pursuing the same plan actively for three-quarters of an hour, animation was perfectly restored.

The next was a case of presentation of the funis; and, as the labour was slow, the child was still-born, but recovered by the same means in half an hour.

The last case with which I shall trouble you was such as to encourage the attempt at resuscitation under any circumstances. It was a case of twins, and the second child presented with the head, before which a considerable portion of the funis had descended. The delivery was extremely slow, from the general weakness of the woman, who had been for a long time in a bad state of health, and the child was born apparently quite dead. As the mother's situation was extremely critical, more than half an hour had elapsed before I could attend to the child; and, on inquiry, I found it had been wrapped in a cloth, and placed on a chair in another room. I immediately made the attempt to restore it, and, by persevering steadily for twenty-five minutes, I had the satisfaction to see symptoms of returning life, and in about fifteen minutes more the child breathed freely.

Every thing in this last case was unfavourable to the restoration of the child,—the mother's long-continued disease, the circumstance of her having two children, and more particularly the delay which took place before any attempt was made, during which time the child was exposed in a room without fire in the winter, with a partial and very slight covering.

I am warranted by my own experience in recommending the attempt to restore all still-born children who have been alive during the birth; and, if the means of resuscitation above mentioned be actively employed and steadily persevered in, I believe the majority of cases will be successful. In all cases the restoration of a child is a most satisfactory circumstance, and in some instances of the greatest possible consequence. I have never found any thing necessary but the regular inflation of the lungs, which I do with my own mouth, in the way I have described, and have generally observed the first symptom of returning life to be a tremulous motion of the respiratory organs; the child next makes a very feeble attempt to inspire, and the colour of the face changes. The inflation should then be made quicker; and, as the attempts to breathe increase, a little sal volatile or brandy, rubbed over the palm of the hand, and held over the mouth during the inflation of air, will materially assist the recovery, and has a better effect than pouring stimulants into the stomach. A few smart slaps on the glutei muscles will now generally complete the recovery.*

*Bridgewater; June* 1827.

* In the Collectanea of the preceding Number, the reader will find a case detailed, in which the life of a child who had taken a large dose of laudanum was saved, by keeping up artificial respiration.

